# Effect of GSE Addition in GICs on Its Adhesion to Bovine Dentin

**DOI:** 10.1155/ijod/4085710

**Published:** 2026-01-22

**Authors:** Allan Inácio Ferreira Piauilino, Sandrine Bittencourt Berger, Eloisa Aparecida Carlesse Paloco, Vitória Fraga Fogaça Melo e Silva, Murilo Baena Lopes, Delise Pellizzaro, Andreza Maria Fábio Aranha, Ricardo Danil Guiraldo

**Affiliations:** ^1^ Department of Dental Sciences, School of Dentistry, University Anhanguera – UNIC, Cuiabá, Mato Grosso, Brazil; ^2^ Department of Restorative Dentistry, School of Dentistry, University Anhanguera – Uniderp, Campo Grande, Mato Grosso do Sul, Brazil; ^3^ Department of Restorative Dentistry, School of Dentistry, University Anhanguera –UNOPAR, Londrina, Paraná, Brazil; ^4^ Department of Restorative Dentistry, School of Dentistry, State University of Londrina, Londrina, Paraná, Brazil, uel.br

## Abstract

**Objectives:**

The aim of the present study was to evaluate microshear bond strength between bovine dentin and glass ionomer cement (GIC) modified with the addition of grape seed extract (GSE).

**Materials and Methods:**

Twenty‐four crowns of bovine incisor teeth were fixed with self‐curing acrylic resin, leaving the buccal surface of the teeth exposed. The enamel surface was removed with SiC abrasive paper, exposing the dentin. Four transparent cylindrical matrices (1 mm high and 0.75 mm in internal diameter) were placed on the dentin surfaces of each tooth. The control groups were composed of the GICs Maxxion R and the GC Gold Label 9 R used in the proportion recommended by the manufacturers. For the experimental groups, GSE was added to the GIC liquid in the form of 10% glycolic extract. The set was stored at an absolute humidity of 37°C for 24 h. After storage, matrices were removed, and specimens were submitted to the microshear bond test.

**Statistical Analysis:**

Data were submitted to two‐way ANOVA and Tukey’s test (*α* = 0.05).

**Results:**

The mean bond strength values did not exhibit statistically significant differences for different groups. For the independent factor material, the average bond strength values were significantly higher for GIC GC Gold Label 9 R (6.15 MPa) when compared to Maxxion R (3.93 MPa).

**Conclusion:**

The incorporation of GSE into GIC did not negatively interfere with the adhesive properties of the GIC; the prevalence of the adhesive failure pattern was observed in all tested groups.

## 1. Introduction

With the advent of the minimal intervention and maximum structure prevention approach to treatable structures, atraumatic restorative treatment (ART) emerged with the purpose of removing infected carious tissue with manual instruments [1]. The affected tissue layer with a hardened consistency and a reduced number of bacteria is preserved, as it has the capacity for regeneration, and a restoration is then carried out with a cement that contains adhesive properties, such as glass ionomer cement (GIC), promoting an adequate environment for the remineralization of affected dentin [1].

GIC has become the material of choice for the ART technique due to its adhesive properties to calcified tooth structures, fluoride release, minimal effect on the dental pulp, and linear thermal expansion coefficient close to dentin [2, 3]. One study has shown that cavities treated with the ART technique may present residual infected dentin with the presence of bacteria, generating, in the short and long term, a failure in the restorative treatment [4].

GIC restorations performed better than amalgams in terms of caries recurrence but had similar results to composite resin restorations [5]. For this reason, the addition of components that could improve their anticariogenic properties without compromising their physical or mechanical properties is timely. To improve the antibacterial properties of GIC, grape seed extract (GSE) was selected, since it exhibits low toxicity and proven activity against several bacteria, including *Streptococcus mutans*, making it a promising alternative to enhance GIC performance for use in the ART technique [6, 7]. However, the addition of antimicrobial agents commonly affects the physical and mechanical properties of the GIC, reducing its resistance to compression, surface hardness, and adhesion [8]. Thus, the aim of the present study was to evaluate microshear bond strength between bovine dentin and GIC or GIC modified with the addition of GSE. The null hypothesis tested is that the addition of GSE to GIC does not have a negative effect on the bond strength of this material to bovine dentin.

## 2. Materials and Methods

### 2.1. Sample Size Calculation

The sample size calculation was performed using Minitab 16 software for Windows 8 (Minitab, State College, PA, USA), selecting the ANOVA test, and was based on data from a previous study [9]. That study reported microshear bond strength with a standard deviation of 2.21. Considering a minimum detectable difference of 3.37, with an alpha error of 0.05 and a beta power of 0.8, the minimum estimated sample size was 11 specimens.

### 2.2. Sample Preparation

Bovine incisor teeth were extracted, cleaned, and stored in 0.1% thymol solution at 4°C until the time of use [10]. Twenty‐four bovine incisor teeth without cracks or enamel defects were selected. The roots were removed with a diamond disc (KG Sorense Ind. Com. Ltda, Barueri, SP, Brazil), and the crown of teeth was included, in a standardized manner, in a PVC tube ring (¾ −1.5 cm) (Odeme Dental Research, Luzerna, SC, Brazil) and fixed with self‐curing acrylic resin (JET Clássico; Artigos Odontológicos Clássico Ltda, São Paulo, SP, Brazil), leaving the buccal surface exposed.

The enamel surface was removed using a polishing machine with #180 grit SiC abrasive paper to expose the flat dentin and prepare the samples. After that, polishing was carried out with SiC abrasive paper in grits of #220, #400, #600, and #1200, in that order, with a predetermined time of 20, 30, 30, and 60 s, respectively. Then, to standardize the smear layer, #600 grit SiC abrasive paper was passed again for another 30 s. Samples were submitted to a 10 min ultrasonic bath (Ultrasonic Cleaner, Odontobras, Araraquara, SP, Brazil) in distilled water for abrasive particle removal.

On the dentin surfaces of each tooth, four transparent cylindrical matrices (1 mm in height and 0.75 mm in internal diameter) were positioned at equidistant points (Tygon tubing – TYG‐03, Saint‐Gobain Performance Plastic, Maime Lakes, FL, USA).

The control groups were composed of the GICs Maxxion R (FGM, Joinville, SC, Brazil) and the GC Gold Label 9 R (GC Corporation, Tokyo, Japan) used in the proportion recommended by the manufacturers (resulting in 2 groups). The composition of the GICs used is described in Table 1. For the experimental groups (resulting in 2 other groups), GSE (Mapric Produtos Farmacocosméticos Ltda, São Paulo, SP, Brazil) was added to the GIC liquid in the form of 10% glycolic extract with a graduated micropipette, resulting in a solution with 90% liquid GIC and 10% GSE (this concentration was previously defined in a pilot study, which tested concentrations of 5%, 7.5% 10%, 12.5%, and 15%). Moreover, according to the study by El Adawi et al. [7], this concentration is below the toxic dose to tissues.

**Table 1:** Composition of the GICs used in this study.

Material	Composition
Maxxion R (FGM, Joinville, SC, Brasil)	Powder: fluoro‐alumino‐silicate glass, and calcium fluoride Liquid: polyacrylic acid, tartaric acid, distilled water
GC Gold Label 9 R (GC Corporation, Tokyo, Japan)	Powder: fluoro‐alumino‐silicate glass, polyacrylic acid powder Liquid: distilled water and polyacrylic acid

The matrices were stabilized on the tooth by a trained, calibrated operator with previous experience in microshear testing [11]. Then, the GICs were manipulated according to the manufacturer and inserted into the matrices with an exploration probe no. 5 (Hu‐Friedy, Chicago, IL, USA). In each tooth, using the same GIC, two matrices were filled with GIC without the addition of GSE and two with the addition of GSE, with a total of four matrices per tooth (thus, 4 groups (*n* = 12) were formed: 1‐ Maxxion R handled in accordance with the manufacturer’s instructions; 2‐ Maxxion R handled with the addition of 10% GSE to the GIC liquid; 3‐ GC Gold Label 9 R handled in accordance with the manufacturer’s instructions; 4‐ GC Gold Label 9 R handled with addition of 10% GSE to the GIC liquid). After this step, the set was stored at an absolute humidity of 37°C for 24 h, and subsequently, the matrices were removed with a scalpel blade, exposing the four cement cylinders with a bond area of 0.44 mm^2^ each. The calculation of the bond area was carried out using the formula a = *π* × *r* ^2^. Where a is the area and *r* is the radius. Thus, 0.375^2^ × *π* = 0.44 mm^2^.

### 2.3. Microshear Test

Subsequently, these cylinders were subjected to a microshear bond test. Thus, the set were fixed to a universal testing machine (DL2000; Emic, São José dos Pinhais, PR, Brazil), and load was applied to the base of the cylinders by using steel wire (diameter 0.2 mm) at a speed of 0.5 mm/min until fracture to determine the microshear bond strength [11]. Bond strength values were calculated, and data expressed in MPa. Each group included 12 teeth, and the same tooth was used for 2 different groups. On the same tooth, using the same GIC, two matrices were filled with GIC without the addition of GSE and two with the addition of GSE. Each tooth had two cylinders per group, providing a total of 24 cylinders per group. The mean for the two cylinders of each tooth was considered as 1 specimen.

### 2.4. Failure Pattern Analysis

After failure, cylinders were observed under optical microscopy (Olympus Corp., Tokyo, Japan) at 40× magnification, and failures were quantitatively classified as: adhesive (failure between dentin and GIC interface), cohesive (failure in the GIC), and mixed (combination of adhesive and cohesive failures).

### 2.5. Statistical Analysis

Statistical analyses were performed in the Minitab 16 software for Windows 8 (Minitab, State College, PA, USA). Data were analyzed through the Kolmogorov–Smirnov normality test, two‐way ANOVA (factors: material, GSE), and Tukey’s test, at a 5% significance level (*α* = 0.05).

## 3. Results

The mean bond strength values did not exhibit statistically significant differences for different groups (*p* = 0.954; Table 2) or for the independent factor GSE (*p* = 0.653; Table 3).

**Table 2:** Mean values of microshear bond strength (MPa) for the different groups.

GIC	Microshear (MPa)	
	Without addition of GSE	With addition of GSE
GC Gold Label 9 R	6.09 (0.73)	6.21 (1.52)
Maxxion R	3.85 (0.40)	4.02 (0.33)

*Note:* There was no statistically significant difference for the Material x GSE combination (*p* = 0.954 Tukey’s test). The standard deviation is given in parenthesis.

Abbreviations: GIC, glass ionomer cement; GSE, grape seed extract.

**Table 3:** Mean values of microshear bond strength (MPa) for the independent factor GSE.

GSE	Microshear (MPa)
Without addition	4.97 (1.48)
With addition	5.11 (1.61)

*Note:* There was no statistically significant difference for the independent factor GSE (*p* = 0.653; Tukey’s test). The standard deviation is given in parenthesis.

Abbreviation: GSE grape seed extract.

As shown in Table 4 (*p*  < 0.001), the average bond strength values were significantly higher for GIC GC Gold Label 9 R (6.15 MPa) when compared to Maxxion R (3.93 MPa).

**Table 4:** Mean values of microshear bond strength (MPa) for the independent factor Material.

Material (GIC)	Microshear (MPa)
GC Gold Label 9 R	6.15 (1.40) A
Maxxion R	3.93 (0.52) B

*Note:* Mean values followed by different uppercase letters are significantly different (*p*  < 0.001; Tukey’s test). The standard deviation is given in parenthesis.

Abbreviation: GIC, glass ionomer cement. GSE, grape seed extract.

After the microshear bond strength test, the samples showed a predominance of adhesive failure patterns in all groups (Table 5).

**Table 5:** Failure type distribution based on Material and GSE (%).

GIC	Adhesive	Mixed	Cohesive
GC Gold Label 9 R without GSE	14 (82,3%)	3 (17,6%)	0 (0,0%)
GC Gold Label 9 R with GSE	22 (95,6%)	1 (4,35%)	0 (0,0%)
Maxxion R without GSE	14 (87,5%)	2 (12,5%)	0 (0,0%)
Maxxion R with GSE	15 (88,2%)	2 (11,7%)	0 (0,0%)

Abbreviations; GIC, glass ionomer cement. GSE, grape seed extract.

## 4. Discussion

GSE possesses bactericidal activity due to its polyphenolic compounds, primarily proanthocyanidins and flavonoids. The mechanism of action is multifaceted and mainly involves the destabilization of the bacterial cell membrane and interference in essential metabolic processes. With the purpose of improving the restorative material’s antibacterial property without altering its adhesive properties, the present study evaluated the adhesion of GIC incorporated with GSE to bovine dentin. For this reason, two chemically activated GICs were tested: GC Gold Label 9 R and Maxxion R, comparing them to the Maxxion R and GC Gold Label 9 R GICs incorporated with 10% GSE. With the purpose of improving the restorative material’s antibacterial property without altering its adhesive properties, the present study evaluated the adhesion of GIC incorporated with GSE to bovine dentin. For this reason, two chemically activated GICs were tested: GC Gold Label 9 R and Maxxion R, comparing them to the Maxxion R and GC Gold Label 9 R GICs incorporated with 10% GSE. To choose these materials, a previous study was used [12], in which 21 experts on GICs evaluated the results of tests on mechanical and optical properties of 18 different brands of restorative GICs, showing that seven brands were below the thresholds for restorative indications and 11 were adequate for this purpose. Thus, the present study chose one GIC among those that were suitable for restoration and another among those that were not suitable for restoration.

The result of this study showed that the addition of 10% GSE did not influence the adhesion properties of the GIC in the immediate test, as there was no loss of bonding strength in the two cements tested (Table 2). In contrast, a previous study showed that the addition of antibiotics, at concentrations of 3% and 4.5%, made a significant difference in reducing compressive strength and adhesion to dentin [13]. Another study showed that the addition of chlorhexidine (CHX) presented an increased setting time, a decrease in surface hardness, and a decrease in bond strength with higher concentrations of CHX [14]. Thus, the incorporation of GSE in GIC liquid does not appear to negatively interfere with the immediate bond strength to bovine dentin, making it a material of choice to add antibacterial properties to GIC in the future.

A previous study with propolis showed a decrease in the flexural and bond strength of the GIC [15]. This factor was also not verified when evaluating the difference between the GICs for the independent factor GSE (Table 3), in which it is only compared whether there is GSE in the GIC liquid or not, with no statistically significant difference also being observed for this factor. Thus, the addition of GSE does not appear to alter the adhesive properties of GIC. Even with the modification, the material maintained its bonding strength to dentin, as demonstrated by the microshear test. This finding supports the hypothesis that GSE could be incorporated into GIC to provide antibacterial properties without compromising adhesion to dental structures, which would be highly relevant for its application in the ART technique. However, future studies are needed to evaluate other properties, such as antibacterial action, hardness, and setting time, to rectify the limitation of the present study.

High‐viscosity GICs are characterized by containing powder whose particles have smaller dimensions than low‐viscosity GICs and also by having lyophilized acid added to the powder [16]. When comparing the two GICs tested (Table 4), it was observed that GC Gold Label 9 R (high viscosity) presented a significantly higher bonding strength when compared to Maxxion R (low viscosity). A fact already observed in a previous study [12] that reports the Maxxion R as a GIC with properties below the thresholds for restorative indications. This finding suggests that the brand of GIC is an important factor for the success of ART restorations. Even though this trial did not evaluate the cost‐efficacy of those materials, their difference is evident.

Analyzing the failure patterns, a greater prevalence of adhesive failures was observed, in which there was a total detachment of the GIC from the tooth structure, making it possible to verify the bonding strength of each cement to the tooth structure. It was also observed that the control groups exhibited a quantity of mixed failures close to the experimental groups, and it can be understood that the incorporation of GSE did not interfere in the evaluation of bond strength properties of the GIC. Considering that there were no cohesive failures. Moreover, GSE can positively interfere with the demineralization and remineralization process [17, 18] due to its potential as a remineralizing agent for tooth enamel and by inhibiting the growth of other bacteria, in addition to those related to tooth decay, such as *Aggregatibacter Actinomycetemcomitans*, *S. aureus*, *E. coli and Pseudomonas aeruginosa* [19].

Thus, in cavities treated with the ART technique that contain residual infected dentin with remaining bacteria, the use of GSE may represent an advance in antibacterial action, highlighting the clinical relevance of the present study. Under the conditions of this in *vitro* study, the null hypothesis was accepted since the addition of GSE to GIC did not influence the bond strength of this material to bovine dentin.

## 5. Conclusion

Based on the analyzed and discussed results, the following conclusions can be drawn:

1. The incorporation of GSE into GIC did not negatively interfere with the adhesive properties of the GIC.
2. Prevalence of the adhesive failure pattern was observed in all tested groups.

## Author Contributions

Allan Inácio Ferreira Piauilino contributed to conceptualization, data curation, investigation, methodology, project administration, visualization, and writing – original draft. Sandrine Bittencourt Berger contributed to methodology, data curation, formal analysis, and writing – review and editing. Murilo Baena Lopes and Andreza Maria Fábio Aranha contributed to methodology, software, and writing – review and editing. Eloisa Aparecida Carlesse Paloco, Vitória Fraga Fogaça Melo e Silva, and Delise Pellizzaro contributed to methodology and writing – review and editing. Ricardo Danil Guiraldo contributed to conceptualization, investigation, methodology, project administration, resources, supervision, and writing – review and editing.

## Funding

No funding was received for this research.

## Conflicts of Interest

The authors declare no conflicts of interest.

## Data Availability

The data that support the findings of this study are available from the corresponding author upon reasonable request.
